# ASTRA – An alternative approach for the posterior urethra

**DOI:** 10.1590/S1677-5538.IBJU.2022.0164

**Published:** 2022-05-10

**Authors:** Luis Otávio Amaral Duarte Pinto, Luiz Augusto Westin, Katia Simone Kietzer, Patrick Ely Teloken, Luciano Alves Favorito

**Affiliations:** 1 Hospital dos Servidores do Estado do Pará Serviço de Urologia Reconstrutora Belém PA Brasil Serviço de Urologia Reconstrutora, Hospital dos Servidores do Estado do Pará, Belém, PA, Brasil; 2 Universidade do Estado do Rio de Janeiro Departamento de Urologia Rio de Janeiro RJ Brasil Departamento de Urologia, Universidade do Estado do Rio de Janeiro - UERJ, Rio de Janeiro, RJ, Brasil; 3 Universidade do Estado do Pará Departamento de Morfofisiologia Aplicada à Saúde Belém PA Brasil Departamento de Morfofisiologia Aplicada à Saúde, Universidade do Estado do Pará - UEPA, Belém, PA, Brasil; 4 Sir Charles Gairdner Hospital Urology Department Perth Australia Urology Department. Sir Charles Gairdner Hospital, Perth, Australia; 5 Universidade do Estado do Rio de Janeiro Unidade de Pesquisa Urogenital Rio de Janeiro RJ Brasil Unidade de Pesquisa Urogenital, Universidade do Estado do Rio de Janeiro - UERJ, Rio de Janeiro, RJ, Brasil

## Abstract

**Introduction:**

Access represents one of the main challenges in performing posterior urethroplasty (1, 2). Several approaches and tactics have been previously described (3). This video demonstrates the Anterior Sagittal Transrectal Approach (ASTRA), which allows better visualization of the deep perineum (4).

**Materials and Methods:**

Our patient was a 65-year-old man with post radical prostatectomy vesicourethral anastomotic stenosis. He failed repeated endoscopic interventions, eventually developing urinary retention and requiring a cystostomy. We offered a vesicourethral anastomotic repair through ASTRA. The patient was placed in the jackknife position and methylene blue instilled through the cystostomy. To optimize access to the bladder neck, an incision of the anterior border of the rectum is performed. Anastomosis is carried out with six 4-0 PDS sutures. These are tied using a parachute technique, after insertion of a 16F Foley.

**Results:**

The patient was discharged after 72 hours, and the Foley catheter was removed after 4 weeks. There were no access-related complications. Retrograde urethrogram 3 months after surgery confirmed patency of the anastomosis. Upon review 5 months after surgery the patient had urinary incontinence requiring 5 pads/day and was considered for an artificial urinary sphincter.

**Discussion:**

In our series of 92 patients who have undergone reconstructive procedure through ASTRA there have been no cases of fecal incontinence. Two patients with prior history of radiotherapy developed rectourethral fistulas. Urinary incontinence was observed in those patients with stenosis after radical prostatectomy.

**Conclusion:**

This video presents a step-by-step description of ASTRA, an approach that provides excellent visualization to the posterior urethra, representing an alternative access for repair of complex posterior urethral stenosis.
